# Protective Role of mTOR in Liver Ischemia/Reperfusion Injury: Involvement of Inflammation and Autophagy

**DOI:** 10.1155/2019/7861290

**Published:** 2019-11-13

**Authors:** Tao Zhang, Jianrong Guo, Jian Gu, Ke Chen, Huili Li, Jiliang Wang

**Affiliations:** Department of Gastrointestinal Surgery, Union Hospital, Tongji Medical College, Huazhong University of Science and Technology, Wuhan 430022, China

## Abstract

Liver ischemia/reperfusion (IR) injury is a common phenomenon after liver resection and transplantation, which often results in liver graft dysfunction such as delayed graft function and primary nonfunction. The mammalian target of rapamycin (mTOR) is an evolutionarily highly conserved serine/threonine protein kinase, which coordinates cell growth and metabolism through sensing environmental inputs under physiological or pathological conditions, involved in the pathophysiological process of IR injury. In this review, we mainly present current evidence of the beneficial role of mTOR in modulating inflammation and autophagy under liver IR to provide some evidence for the potential therapies for liver IR injury.

## 1. Introduction

Liver resection and transplantation are the most effective approaches for liver cancer and other end-stage liver diseases. However, liver ischemia/reperfusion (IR) injury is a common complication after liver surgery, which is characterized by aggravated hepatocellular damage in the ischemic liver after the restoration of blood flow [1]. Additionally, abdominal trauma, myocardial ischemia, stroke, and hemorrhagic shock can also cause insufficient liver blood flow, resulting in liver IR injury after reperfusion. Liver IR injury can be divided into warm IR injury and cold IR injury, based on different ischemia conditions. The warm IR injury develops during liver surgery and various forms of shock and trauma, while the cold IR injury occurs during liver transplantation [2]. The severity of the injury ranges from moderate serum aminotransferase level increase to postoperative liver failure after liver resection or to delayed graft function and even primary nonfunction after liver transplantation [3]. Thus, it is of vital importance to investigate the underlying mechanisms and search for possible interventions to protect the liver from IR injury.

Various factors are involved in the pathophysiological process of liver IR injury, including active oxygen species (ROS) overproduction, excessive inflammatory response (redundant inflammatory cytokine release and activation of complement system), the overactivation of autophagy and endoplasmic reticulum stress (ERS), and mitochondrial dysfunction [2]. Among all these factors, inflammation and autophagy are two critical ones. Mammalian target of rapamycin (mTOR) is a critical regulator of cell growth and metabolism that senses and integrates various signals under physiological and pathological conditions, playing critical roles in regulating liver IR injury [4–9].

In this review, we will focus on the role of mTOR signaling in regulating inflammation and autophagy processes in liver IR injury, highlighting the protective role of mTOR signaling and providing some evidence for the potential therapies for liver IR injury.

## 2. mTOR Signaling Pathway

The mammalian target of rapamycin (mTOR) is an evolutionarily highly conserved serine/threonine protein kinase that plays a vital role in regulating mRNA translation, metabolism, and protein turnover [10]. And its dysfunction relates to autoimmune diseases, cancer, obesity, and senescence [11]. mTOR combines with several proteins to constitute two distinct complexes, named mTOR complexes 1 (mTORC1) and 2 (mTORC2). mTORC1 is composed of five components: mTOR, regulatory protein associated with mTOR (Raptor), mammalian lethal with Sec13 protein 8 (mLST8 or GßL), proline-rich Akt substrate of 40 kDa (PRAS40), and DEP domain containing mTOR interacting protein (DEPTOR). mTORC2 is composed of mTOR, rapamycin insensitive companion of mTOR (Rictor), mLST8, DEPTOR, and the regulatory subunits mSin1 and Protor1/2 [10]. mTORC1 integrates stimuli from intracellular and extracellular cues, such as growth factors, energy status, amino acids, stress, and oxygen, and is sensitive to rapamycin. mTORC1 plays a crucial role in controlling protein, lipid, nucleotide, and glucose metabolism, autophagy, energy metabolism, lysosome biogenesis, cell survival, and cytoskeletal organization [12]. mTORC2 is insensitive to nutrients and acute rapamycin treatment but sensitive to growth factors [12], which regulate cell cytoskeletal remodeling, cell migration, glucose metabolism, ion transport, and cell survival [10]. Moreover, mTORC2 can phosphorylate and activate Akt (on S473), a major effector of the insulin/PI3K pathway, which is essential for the activation of mTORC1 [10]. Besides, mTORC2 can also be phosphorylated and activated by Akt in the subunit of mSin1 (on T86) [13]. Since mTORC1 is the better characterized and well-studied mTOR complex and exerts major regulatory function on various fundamental cell processes, we will mainly focus on mTORC1 in this review.

**Figure 1:**
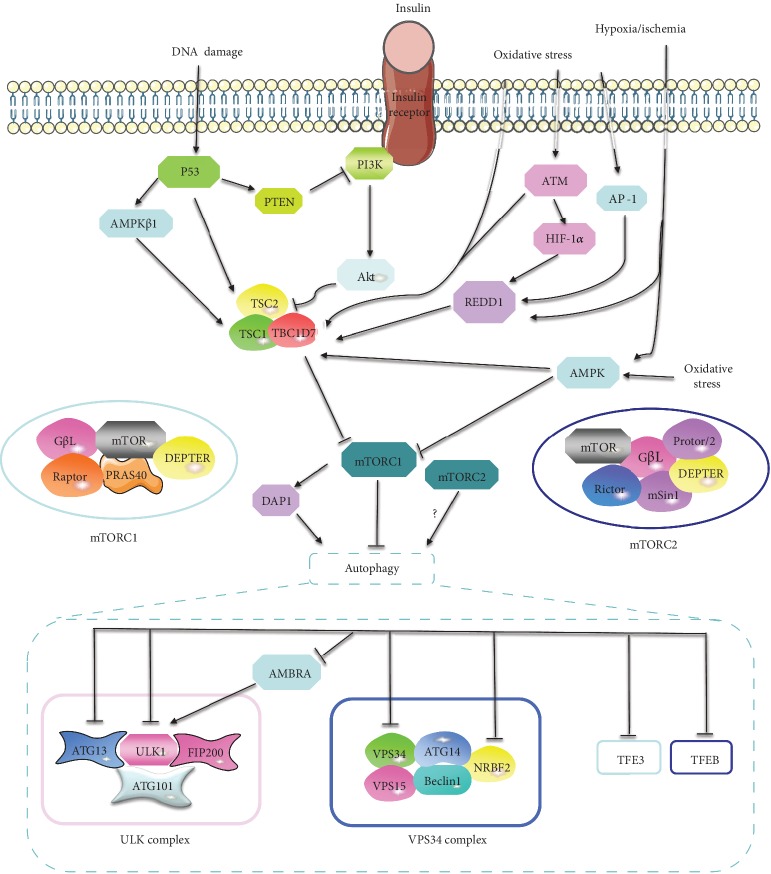
The mTOR signaling pathway is involved in liver IR injury and plays crucial roles in autophagy. Growth factors such as insulin activates mTORC1 through the PI3K/Akt/mTORC1 pathway. However, the activation of the AMPK signaling pathway will lead to the inhibition of mTORC1 through activating TSC complex. Hypoxia/ischemia, oxidative stress, and DNA damage are mechanisms commonly involved in liver IR injury. The decrease of ATP induced by hypoxia/ischemia activates AMPK, which inhibits mTORC1 through activating TSC or suppressing mTOR directly. Additionally, hypoxia/ischemia also activates REDD1, which promotes the TSC-mediated suppression of mTOR. Oxidative stress induces the activation of ATM, which inhibits mTORC1 through activating TSC directly or through phosphorylating HIF1*α*, resulting in induction of REDD1, causing the activation of TSC. Besides, oxidative stress promotes the activation of AP-1, which transcriptionally upregulates the expression of REDD1. Finally, DNA damage inhibits mTOR through inducing PTEN, AMPK*β*1, and TSC, which are targeted by p53. mTOR signaling plays a crucial and complex role in autophagy. In the initial phase of autophagy, mTORC1 inhibits ULK1 complex (ULK1/Atg13/ATG101/FIP200) via directly phosphorylating ULK1 and ATG13. Besides, mTORC1 can also inhibit ULK complex through phosphorylating and suppressing AMBRA, which enhances the activity and stability of ULK1. Additionally, mTORC1 represses the initial of autophagy also through inhibiting VPS34 complex (VPS34/VPS15/Beclin1/ATG14/NRBF2) by directly phosphorylating ATG14 and NRBF2. In the elongation/closure phase, mTORC1 suppresses autophagic and lysosomal biogenesis through phosphorylating TFEB and TFE3 to modulate their nuclear-cytoplasmic shuttling. Moreover, mTORC1 can also augment autophagy through phosphorylating DAP1, which acts as a buffering mechanism that counterbalances the autophagic flux and prevents its overactivation. Additionally, mTORC2 also participated in the induction of autophagy through an unclear mechanism.

mTORC1 integrates upstream signaling molecules such as growth factors (insulin), epidermal growth factor (EGF), amino acids, energy, stress, and mitogens via multiple signaling pathways [14]. There exist four major upstream signaling pathways of mTORC1, including the insulin/phosphatidylinositol-3 kinase/protein kinase B (insulin/PI3K/Akt) signaling pathway, EGF/Ras/Raf/mitogen activated protein kinase (EGF/Ras/Raf/Mek/Erk) signaling pathway, Wnt/glycogen synthase kinase-3*β* (Wnt/GSK-3*β*) signaling pathway, and adenosine monophosphate-activated protein kinase (AMPK) signaling pathway [12, 15]. All of these four axes are converged at least partially on tuberous sclerosis complex (TSC), which is composed of TSC1, TSC2, and TBC1D7 and functions as a GTPase activating protein (GAP) of the Ras homolog enriched in brain (Rheb) GTPase. The GTP-bound form of Rheb directly binds and activates mTORC1 activity. As a GAP of Rheb, TSC converts GTP-Rheb into its inactive GDP-bound form to inhibit the activity of mTORC1 [10] (Figure 1).

## 3. The mTOR Signaling and Liver IR

### 3.1. Inhibition of mTOR Signaling in Liver IR

The inhibition of mTOR signaling during liver IR has been shown in many studies [5, 6, 9]. Hypoxia/ischemia, oxidative stress, and DNA damage are commonly involved in liver IR injury [16], which suppress mTOR through various molecular pathways.

Under conditions of hypoxia/ischemia, liver AMPK is activated in a very short window of time (about 2 min) to respond to the increased intracellular AMP/ATP and/or ADP/ATP ratio [17, 18]. Activated AMPK suppresses mTORC1 by phosphorylating TSC (on S1345) to amplify the inhibitory activity of TSC to mTORC1 [19]. Besides, AMPK directly phosphorylates Raptor (on S792), a component of mTORC1, leading to the allosteric inhibition of mTORC1 [19]. In addition, hypoxia/ischemia inhibits mTORC1 also through mediating regulated in DNA damage and development 1 (REDD1) in hepatocytes [20]. In response to hypoxia/ischemia, the expression of REDD1 is transcriptionally upregulated [21]. REDD1 converges on TSC and promotes TSC-mediated suppression of mTORC1 through mediating 14-3-3 protein shuttling from TSC to REDD1 [15].

Oxidative stress, known as redox balance dysregulation and overformation of ROS, also exerts inhibitory effects on mTORC1 [22–28]. Antioxidants such as N-acetylcysteine [29] and hydrogen sulfide [8, 30] can effectively restore the activity of mTORC1, which is repressed by oxidative stress in organ IR injury, including the liver. The mechanisms behind may be as follows: ROS can activate TSC to suppress the activation of mTORC1 [31]. Besides, ROS also inhibits mTORC1 through activating cytoplasmic ataxia telangiectasia mutated (ATM) [22, 25]. Activated ATM further activates TSC [22] or phosphorylates HIF1*α*, leading to the activation of REDD1 [32], resulting in the inhibition of mTORC1. Additionally, H_2_O_2_-induced ROS burst can induce the activation of activator protein-1 (AP-1), which transcriptionally regulates the activation of REDD1 in hepatocytes [33], leading to the suppression of mTORC1. Moreover, ROS can inhibit mTORC1 through activating AMPK as well [24, 34].

Finally, the DNA damage will lead to the activation of p53, which causes the activation of TSC2, phosphatase and tensin homolog deleted on chromosome 10 (PTEN), and *β*1 subunits of the AMPK (AMPK*β*1), resulting in the suppression of mTORC1 [35] (Figure 1).

### 3.2. The Beneficial Effects of mTOR Signaling in Liver IR Injury

The beneficial effects of mTOR in IR have been observed in the heart [36–43], brain [44–47], intestine [48, 49], and kidney [50]. Similarly, the protective function of mTOR signaling in liver IR injury has been revealed in some studies (Table 1). Bortezomib [4], melatonin [5], geniposide [7], NaHS [8], and agomir-miR-494 [9] administration attenuated liver IR injury through activating mTOR signaling. Additionally, genetic overexpression of liver mTOR directly significantly reduces liver inflammation and apoptosis induced by IR [6].

In this review, we focused on the impact of mTOR signaling on inflammatory response and autophagy to discuss the beneficial effect of mTOR signaling on liver IR injury.

## 4. mTOR Attenuates Inflammation Response in Liver IR Injury

An excessive inflammatory response is recognized as a key mechanism of liver IR injury. Inflammatory networks, including inflammatory cells and humoral factors, play a vital role in liver IR injury [51]. Kupffer cells (KCs), neutrophils, CD4^+^ T lymphocytes, and natural killer T (NKT) cells are the main cellular participants. Complement factors, cytokines, and chemokines are the main humoral factors. Additionally, sinusoidal endothelial cells (SECs) and hepatocytes are also important participants and play critical roles in the inflammatory response during the liver IR process, leading to hepatocellular damage [52].

**Figure 2:**
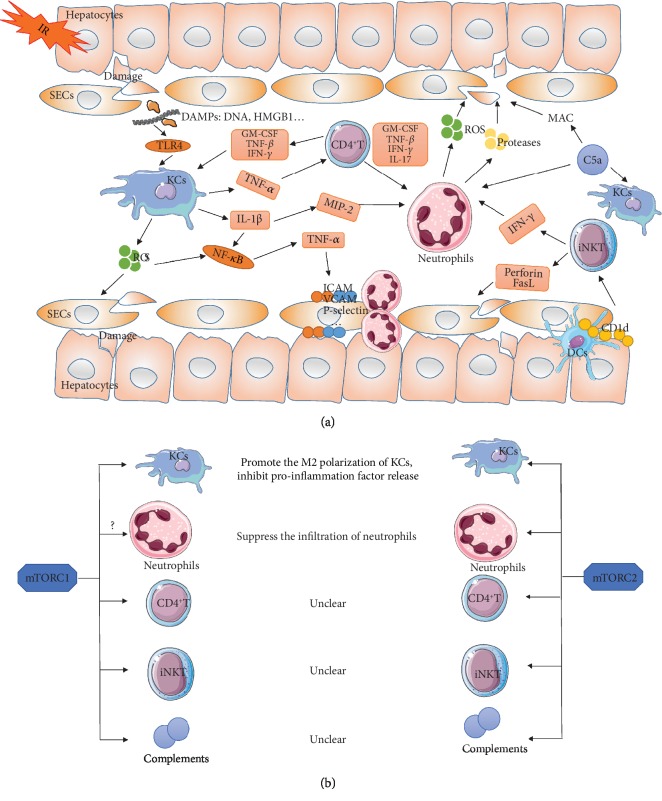
(a) Schematic diagram of the inflammatory response during liver IR injury. Liver IR injury induces the damage of hepatocytes and SECs, leading to the release of DAMPs, resulting in the activation of KCs. Activated KCs release ROS and proinflammatory molecules (TNF-*α*, IL-1*β*), leading to the injury of hepatocytes and SECs and the activation of neutrophils and CD4^+^ T lymphocytes. The activation of CD4^+^ T lymphocytes amplifies the activation of KCs and neutrophils through releasing GM-CSF, TNF-*β*, and INF-*γ*. Activated neutrophils lead to the damages of hepatocytes and SECs through the release of ROS and proteases. iNKT cells are activated through interacting with CD1d, expressing on hepatocytes and APC within the liver. Activated NKT cells damage the liver directly through secreting perforin and FasL and through activating neutrophils. The complement system is activated in IR injury, which induced cell lysis via the formation of MAC or through activating KCs and neutrophils. (b) The impact of mTOR signaling on inflammatory response in liver IR injury. During liver IR injury, both mTORC1 and mTORC2 promote the M2 polarization of KCs (macrophages) and inhibit the release of proinflammation factors. Besides, mTORC2 also suppresses the infiltration of neutrophils during liver IR injury. Additionally, mTORC1 may play a role in inhibiting neutrophil infiltration through negatively regulating ICAM-1 expression in SECs. However, the role of mTOR signaling on CD4^+^ T lymphocytes, iNKT, and the complement system in liver IR injury remains unclear.

The mTOR signaling is appreciated to be a potent activator of the immune response, as its role in regulating cellular metabolism which is closely related to the proliferation and activation of immune cells, including neutrophils, mast cells, macrophages, dendritic cells (DCs), T lymphocytes, and B lymphocytes [53, 54]. However, an increasing body of evidence has emerged indicating that mTOR plays a pivotal anti-inflammatory role in liver IR injury. Myeloid mTOR activation through PTEN deficiency leads to the suppression of liver immune activation and protected livers from IR injury [55]. Additionally, mTOR-deficient mice showed greater expression levels of inflammation-related genes such as MCP-1, TNF-*α*, and IL-6 than wild-type (WT) mice after liver IR via negatively modulating NF-*κ*B [6]. Besides, the opposite effect was seen in TSC1-deficient (mTOR-activated) mice, which showed a weaker inflammation response to liver IR injury than WT mice [6]. However, the mechanism of mTOR signaling in regulating the inflammatory response in liver IR injury largely remains unclear. In this section, we will discuss the regulatory role of mTOR signaling on inflammatory cells and humoral factors in liver IR injury (Figure 2).

### 4.1. Kupffer Cells (KCs)

KCs, the liver-resident macrophages, play a key role in initiating and propagating inflammatory response of liver IR injury. In the early stage of reperfusion (within 2 h), KCs are activated by damage-associated molecular patterns (DAMPs), such as high-mobility group box 1 protein (HMGB1) and DNA fragments, through activating Toll-like receptor 4 (TLR4) [56]. On activation, KCs release ROS and proinflammatory molecules, including IL-1*β* and TNF-*α*. ROS induces oxidative damages to proteins, enzymes, nucleic acids, cytoskeleton, and lipid, leading to mitochondrial dysfunction and lipid peroxidation, contributing to injury of hepatocytes and SECs, resulting in both apoptotic and necrotic cell death [57]. ROS can also activate NF-*κ*B, which upregulates the expression of proinflammatory cytokines, including TNF-*α* [58]. IL-1*β* activates NF-*κ*B and macrophage inflammatory protein-2 (MIP-2), leading to the aggregation and adhesion of neutrophils [59]. TNF-*α* recruits and activates neutrophils and CD4^+^ T lymphocytes to the site of injury [60].

Numbers of studies have revealed the impact of mTOR signaling on KCs (or macrophages). The inhibition of mTORC1 increases inflammation and promotes the recruitment of inflammatory macrophages by enhancing NF-*κ*B activity [61]. The Akt/mTOR signaling pathway can convert proinflammatory M1 macrophage into the anti-inflammatory M2 type through regulating the expression and phosphorylation of Acly [62]. Acly is a key enzyme in Ac-CoA synthesis, which increases the production of Ac-CoA in M2 macrophages and leads to the activation of M2 macrophages, resulting in the suppression of inflammation [62]. Besides, the Akt/mTOR signaling pathway can also integrate metabolic signals to support the activation of M2 macrophage [62]. However, researchers also found that increased activity of mTORC1 by ablating TSC1 promoted M1 macrophage polarization and suppressed M2 macrophage polarization [63]. The controversial results may be due to the fact that macrophage polarization is also regulated by environmental cues. And the different environmental and metabolic cues sensed by mTOR signaling influence macrophage polarization in some complex and unknown manners [64]. In the context of liver IR injury, astaxanthin activated the Akt/mTOR/HIF-1*α* signaling pathway in KCs, reducing the production of ROS and the expression of inflammatory cytokines, attenuating liver IR injury [65]. Similarly, activation of mTORC1 induced by PTEN deficiency promotes the M2 polarization of macrophages and increases the production of IL-10, decreasing the release of TNF-*α*, IL-6, and IL-12 when responding to TLR stimulation in liver IR injury [66]. Additionally, the deficiency of Rictor, a core component of mTORC2, increases the infiltration of macrophage/neutrophil and the release of cytokine/chemokine during liver IR injury [67], indicating an important role of mTORC2 on suppressing KCs.

### 4.2. Neutrophils

The activation of neutrophils is the major cause of injury in the late phase of liver IR (between 6 and 24 h after reperfusion) [68]. As described above, during the first 2 h of reperfusion, KCs activate and release ROS and proinflammatory cytokines, including TNF-*α*, which upregulates intercellular adhesion molecule-1 (ICAM-1), vascular cell adhesion molecule-1 (VCAM-1), and P-selectin on the surface of SECs and hepatocytes, leading to the accumulation of neutrophils in the sinusoidal space and causing microcirculatory disturbances [69]. Additionally, neutrophils migrate toward the site of injury through extravasation and chemotaxis [70]. The accumulation of neutrophils in the site of injury leads to hepatocellular damages through degranulation with release of a large amount of proteases and ROS [71]. Additionally, neutrophils propagate the inflammatory response by recruiting other members of the immune system [72].

The mTOR signaling plays critical roles in the proliferation and activation of neutrophils [53, 54]. Rapamycin promotes the infiltration of neutrophils through inducing the expression of ICAM-1 via the activation of NF-*κ*B in endothelial cells, indicating that mTORC1 can inhibit the migration of neutrophils [73]. Besides, in the liver [67] and kidney [74] IR injury, mTORC2 suppresses the infiltration of neutrophils and attenuates organ IR injury. However, the role and mechanism of mTOR signaling (especially mTORC1) on regulating neutrophils in IR injury remain unclear, and further studies are needed to investigate.

### 4.3. CD4^+^ T Lymphocytes

CD4^+^ T lymphocytes are important cellular participants of inflammation response in liver IR injury, which plays a critical role in promoting liver IR injury [75–77]. As described above, the activation of KCs can activate CD4^+^ T lymphocytes through releasing TNF-*α*. Activated CD4^+^ T lymphocytes release granulocyte-macrophage colony-stimulating factor (GM-CSF), TNF-*β*, and INF-*γ*, which in turn amplify the activation of KCs and promote the recruitment of neutrophils into the liver sinusoids [78, 79]. What is more, CD4^+^ T lymphocytes can also recruit neutrophils through producing IL-17 [80].

mTOR signaling is crucial for the differentiation of CD4^+^ T lymphocytes [81]. However, the role and mechanism of mTOR signaling on regulating CD4^+^ T lymphocytes in liver IR injury remain unclear; further investigations are needed to reveal.

### 4.4. Natural Killer T (NKT) Cells

Natural killer T (NKT) cells are a kind of markedly enriched nonconventional T cells in the liver, accounting for up to 30% of the intrahepatic lymphocytes [82]. NKT cells are divided into two subtypes: Type I (invariant, iNKT) and Type II; iNKT accounts for the majority [83]. The high abundance of iNKT cells in the liver and their rapid response (within hours) to activation suggest that they might play a role in liver IR injury [83, 84]. iNKT cells are recruited to the postischemic liver and are activated through interacting with CD1d antigen-presenting molecules, which express on hepatocytes and antigen-presenting cells (APC) within the liver. Activated iNKT cells damage the liver directly through secreting perforin and FasL and indirectly through activating neutrophils by the production of IFN-*γ* [85, 86]. Reducing the recruitment and cytokine production of iNKT cells ameliorates liver IR injury [87, 88]. Contrary to iNKT cells, Type II NKT cells have an anti-inflammatory effect [89].

Several studies have demonstrated the importance of mTOR in iNKT cells. mTOR signaling plays a critical role for both early and late stages of iNKT cell development [90]. However, the role and mechanism of mTOR signaling in mediating iNKT cells in liver IR injury remain unknown; further investigations are thus needed to explore.

### 4.5. Complement System

Apart from the immune cells, cytokines, and chemokines mentioned above, the complement system serves as an important participant of the inflammatory response in liver IR injury. The complement system consists of about 30 soluble and membrane-bound proteins, which are a well-acknowledged mediator of inflammation. Studies have shown that IR activated the complement system through the classical, the alternative, or the mannose-binding lectin (MBL) pathways [91]. On activation, the complement system induces cell lysis via the formation of membrane attack complexes (MAC). Additionally, the activated complement factor, C5a, can also activate KCs and recruit and activate neutrophils [92, 93], leading to liver damage. Additionally, complement system inhibitors, including C5a receptor (C5aR) antagonist, C5a monoclonal antibodies, C1 inhibitor, cobra venom factor (CVF), and soluble complement receptor type 1 (sCR1), have been shown to be effective in attenuating liver IR injury [94].

Researches revealed that the complement system linked tightly with mTOR signaling [95]. In the CD4^+^ T lymphocytes, C3a activates the C3aR on lysosomes, causing low-level mTOR activation, and C3a binding to C3aR on the cell surface results in sustained mTOR activity. Besides, C3b also associated with mTOR [95]. On the other hand, the activation of mTOR signaling significantly suppresses LPS-induced C5aR expression in macrophages [96]. However, the role of mTOR signaling in mediating the complement system in liver IR injury remains unknown; further investigations are thus needed to explore (Figure 2).

Besides, a recent study found that mTOR signaling played roles in maintaining the activity of CD4^+^Foxp3^+^ regulatory T cells (Tregs), which is capable of modulating other immune cells and suppressing the inflammatory response, indicating a novel mechanism of the anti-inflammatory role of mTOR in IR injury [97].

## 5. mTOR Inhibits Excessive Autophagy in Liver IR Injury

### 5.1. Definition of Autophagy

Autophagy is an evolutionarily highly conserved self-degradative process that targets intracellular components to lysosomes for degradation and recycling to maintaining cellular homeostasis [98]. There are four recognized types of autophagy: macroautophagy, microautophagy, chaperone-mediated autophagy (CMA), and noncanonical autophagy [99]. Here, we will focus on macroautophagy, which will be henceforth referred to as autophagy.

In nutrient-rich conditions, autophagy holds at a low level to maintain intracellular homeostasis through the removal of long-lived and malformed proteins and damaged organelles, called basal autophagy [100]. The activity of cellular autophagy can be markedly upregulated by starvation, hypoxia, energy depletion, ERS, infection, and other stimuli, which are called induced autophagy [101]. Upon induction, small double-membrane vesicles, called autophagosomes, sequester proteins, damaged organelles, and exogenous pathogens. And then, the outer membrane of autophagosomes fuses with lysosomes to form autolysosomes, in which the cargos and inner membrane of autophagosomes were degraded into biological active macromolecules (amino acids, nucleotide, free fatty acids, etc.) and be recycled for the synthesis of protein and ATP [102]. Furthermore, autophagy also plays a pivotal role in cellular homeostasis by regulating the turnover of mitochondria [103], ER [104], peroxisomes [105], and lipid [106] through selective forms. Nevertheless, excessive autophagy, leading to excessive degradation of essential proteins and organelles, can also induce a programmed cell death, called Type II programmed cell death [107]. Additionally, there also exist crosstalks between autophagy and other cell death mechanisms, including apoptosis and necrosis [108].

### 5.2. Autophagy and Liver IR Injury

An increasing body of evidence has emerged indicating that autophagy plays pivotal roles in IR injury of the heart [109], liver [3], brain [110], kidney [111], and lung [112], whereas its role remains controversial in these organs. Recent studies indicated that autophagy acts as a double-edged sword in either a beneficial or a detrimental way in ischemia and reperfusion phases, respectively. Autophagy acts as a compensatory mechanism to counterbalance ATP deprivation in the stage of ischemia, while sustained and excessive activation of autophagy during reperfusion phage results in cell death [113]. It was proven by multiple types of research in heart [114], liver [115], and brain [116] IR injuries. Additionally, a recent study found in the model of hypoxia/reoxygenation of adipose-derived mesenchymal stem cells (ADMSC) that the autophagy of ADMSC activated in the initial hypoxia period and markedly enhanced in the phase of reoxygenation. Interestingly, in the hypoxia phase, apelin upregulated protective autophagy through activating the AMPK/mTOR/ULK1 pathway. In the reoxygenation period, apelin suppressed excessive autophagy through Akt/Bcl2/Beclin1 signaling [117]. Similarly, berberine exerts protective effects in IR injury both through activating autophagy [118] and suppressing excessive autophagy [119]. The dual modulative effects of apelin and berberine keep autophagy activity at a moderate level to be protective for cell survival.

Accumulative evidence has shown that autophagy was hepatoprotective in liver IR injury [120–124]. However, some studies suggested that autophagy was deleterious in liver IR injury [5, 115, 125, 126]. The different results may be attributed to the various magnitudes of autophagy, owing to the different types of IR mode (cold/warm or partial/global IR) and the different liver conditions (lean/fatty), which lead to the different levels of autophagy activation. The controversial results may also owe to the “side effects” (except for regulating autophagy) of interventions adopted in the researches (Table 2).

### 5.3. mTOR Signaling and Autophagy

Autophagy can be regulated by the Bcl-2 signaling pathway, mTOR signaling pathway, MAPK signaling pathway, and p53 signaling pathway [127]. Among them, autophagy is mainly negatively regulated by the mTOR signaling pathway [128].

Under physiology conditions, growth factors such as insulin or EGF activate mTORC1 through insulin/PI3K/Akt/mTORC1 and EGF/Ras/Raf/Mek/Erk/mTORC1 axis, respectively [15]. Activated mTOR signaling exerts a potent inhibitory effect on multiple phases of autophagy [129]. In the initiation phase, mTORC1 suppresses autophagy by inhibiting ULK complex (ULK1/Atg13/ATG101/FIP200), a kinase complex indispensable to initiate autophagy [130] via directly phosphorylating ULK1 (on S317) and ATG13 [131]. Besides, mTORC1 can also inhibit ULK complex through phosphorylating and suppressing Beclin 1-regulated autophagy protein 1 (AMBRA) [132], which enhances the activity and stability of ULK1. Additionally, mTORC1 represses the initial of autophagy also through inhibiting another crucial complex for autophagy induction, vacuolar protein sorting 34 (VPS34) complex (VPS34/VPS15/Beclin1/ATG14/NRBF2), by directly phosphorylating ATG14 and nuclear receptor binding factor 2 (NRBF2) (on S133 and S120) [129]. In the elongation/closure phase, mTORC1 suppresses autophagic and lysosomal biogenesis through phosphorylating TFEB (on S211) and TFE3 (on S321) to modulate their nuclear-cytoplasmic shuttling [129] (Figure 1). Moreover, mTORC1 can also augment autophagy through phosphorylating death-associated protein 1 (DAP1), which acts as a buffering mechanism that counterbalances the autophagic flux and prevents its overactivation [133]. Additionally, researchers found that the activation of mTORC2 also participated in the induction of autophagy [134] (Figure 1).

As shown above, in conditions of hypoxia/ischemia, oxidative stress and DNA damage induced by liver IR, AMPK, REDD1, and ATM will be activated and lead to the suppression of mTOR signaling, resulting in the activation/overactivation of autophagy. Since the indispensable role of mTOR signaling on autophagy, numerous researches focused on mTOR signaling for regulating autophagy to protect against liver IR injury. Researches have shown that melatonin [5] and microRNA-101 [135] attenuated liver IR injury by suppressing autophagy through activating mTOR signaling. However, researches have also shown that activation of autophagy through mTOR inhibitor rapamycin [136–138] and everolimus [121] has been shown to protect against liver IR injury. In addition, a recent study showed that inhibition of mTORC2 by Rictor deficiency aggravated liver IR injury through suppressing autophagy [67]. The paradox result may be attributed to the double-edged effect of autophagy in liver IR injury that the moderate level of autophagy mitigated liver IR injury in ischemia, while the excessive level of autophagy aggravated liver IR injury in reperfusion. This explains the finding that in moderate and advanced steatotic liver that autophagy was impaired, melatonin combined with trimetazidine elevated liver autophagy, rather than inhibited, and improved liver IR injury [139].

These findings indicated an intricate function of autophagy in liver IR injury and a complexity effect of the mTOR pathway in regulating autophagy. We believe that moderate regulation of autophagy through modulating the PI3K/Akt/mTORC1 pathway or mTORC1/mTORC2 balancing may serve as a potential strategy for attenuating liver IR injury.

## 6. Conclusions

Liver IR injury is a clinical phenomenon in various settings including liver resection and transplantation, which is a major cause of morbidity and mortality in liver surgeries and limits the use of grafts available for transplantation.

Liver IR injury is typified by the excessive inflammatory response, which involves a complex interaction network between the inflammatory cells and humoral factors, leading to liver dysfunction and cell injury. Although mTOR signaling is a potent proinflammatory regulator on the growth and differentiation of multiple inflammatory cells, in the context of liver IR injury, it seems to play a significant role in anti-inflammation through regulating KCs and neutrophils. However, the mechanism of mTOR signaling on anti-inflammation still remains unclear, especially on the regulation of CD4^+^ T lymphocytes, NKT cells, and complement systems in the context of liver IR injury.

Additionally, significant changes of autophagy in hepatocytes are observed in liver IR injury; enhancing autophagy under ischemia conditions can promote survival, whereas excessive and long-term augmentation of autophagy during reperfusion may promote cell death. mTOR signaling plays a complexity effect in regulating autophagy. Keeping autophagy at a moderate level during liver IR through modulating the PI3K/Akt/mTORC1 pathway or mTORC1/mTORC2 balancing may serve as a potential strategy for attenuating liver IR injury. A comprehensive study and an illuminating evaluation of the mTOR pathway are thus needed before clinical usage of the autophagy regulator in liver IR patients.

However, in contrast to the beneficial effect of mTOR mentioned above, some studies have shown that berberine precondition [140], isoflurane precondition [141], and rapamycin dealing [138, 142], which associated with inhibition of mTOR signaling, also showed protective effects on liver IR injury, indicating a detrimental role of mTOR signaling in liver IR injury (Table 1). The controversial results may be due to the “side effects” (except for regulating mTOR) of the interventions and the different levels of autophagy in the liver IR models adopted in these researches (Table 1). Besides, Li et al. utilized complementary genetic models with gain or loss of function of mTOR signaling in the liver and demonstrated the beneficial effect of mTOR in liver IR injury [6]. Thus, we hold the idea that mTOR signaling plays a protective role in liver IR injury.

**Figure 3:**
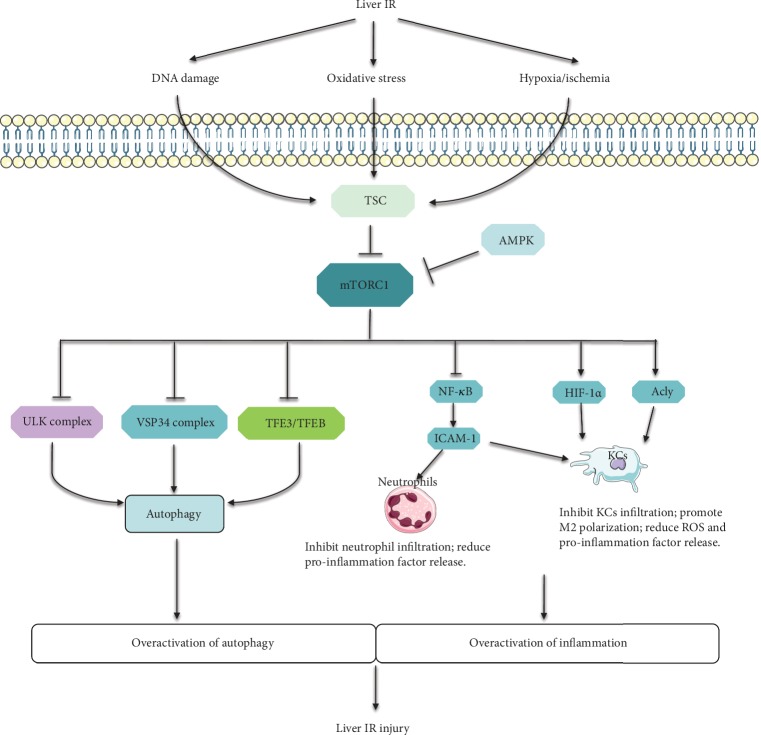
The summary of the protective role of mTOR in liver IR injury: involvement of inflammation and autophagy. During liver IR injury, IR-induced hypoxia/ischemia, oxidative stress, and DNA damage suppress mTORC1 through activating TSC or AMPK via multiple signaling pathways. The repression of mTORC1 leads to the overactivation of autophagy through activating ULK complex, VSP34 complex, and TFE3/TFEB. Additionally, the inhibition of mTORC1 promotes the infiltration of neutrophils and KCs through the NF-*κ*B/ICAM-1 axis. Besides, mTORC1 suppression also reduces the M2 polarization and promotes ROS and proinflammation factor release through inhibiting Acly and HIF-1*α*, resulting in the overactivation of inflammation. The overactivation of autophagy and inflammation leads to the liver IR injury finally.

In a word, the impact of mTOR signaling on the inflammatory response and autophagy provides an attractive therapeutic target for liver IR injury (Figure 3).

## Figures and Tables

**Table 1:** The effect of mTOR in liver IR injury.

Study	Effect of mTOR	Animal model	Interventions	“Side effects” of intervention
Bejaoui et al. [4]	Protective	Obese Zucker rats	Bortezomib (100 nmol/L) addition to Institut George Lopez- (IGL-) 1 preservation solution	Enhances the activity of AMPK [4]. Attenuates inflammatory processes through YKL-40 [143] and NF-*κ*B [144, 145] inhibition. Activates endothelial nitric oxide synthase (eNOS) [146]
Kang et al. [5]	Protective	C57BL/6 mice	Melatonin (10 mg/kg, i.p.) 15 min prior to ischemia and again before reperfusion	Inhibits oxidative stress. Improve the endothelial function. Restores mitochondrial function. Suppresses TLR and JNK pathways [94]. Activates RISK, SAFE, ERK1/2, PKB, PKC, JAK/STAT3, SIRT1/SIRT3, AMPK*α*, and Notch1/Mfn2 pathways [122]
Li et al. [6]	Protective	Alb-TSC1^−/−^ and Alb-mTOR^−/−^ transgenic mice	Overexpression and knockdown of liver mTOR	None
Rong et al. [7]	Protective	Sprague-Dawley (SD) rats	Geniposide (5, 10, and 20 mg/kg, i.p.) 30 minutes before ischemia	Inhibits oxidative stress through activating heme oxygenase-1 (HO-1) [147]. Prevents apoptosis via improving mitochondrial dysfunction and activating glucagon-like peptide-1 receptor (GLP-1R) [148]
Shimada et al. [8]	Protective	C57BL/6J mice	NaHS (1 mg/kg, i.v.) 10 min before reperfusion	Inhibits lipid peroxidation and inflammation reactions. Upregulates intracellular antioxidant and antiapoptotic signaling pathways. Inhibits mitochondrial permeability transition pore (mPTP) opening, reduces cell apoptosis, and activates Akt/GSK3*β* signaling [149]
Su et al. [9]	Protective	Sprague-Dawley (SD) rats	agomir-miR-494 (20 *μ*L of 500 pmol/d, 7 d, i.p.) prior to ischemia	Upregulates hypoxia-inducible factor-1 alpha (HIF-1*α*) and HO-1 [150]. Inhibits proapoptotic protein PTEN, ROCK1, and CaMKII*δ* [151]
Sheng et al. [140]	Detrimental	Sprague-Dawley (SD) rats	Berberine pretreatment (100 mg/kg/d, 2 weeks)	Reduces oxidative stress, inflammation response, endoplasmic reticulum stress (ERS), and apoptosis via activating silent information regulator 1 (SIRT1) signaling [152] and Janus kinase/signal transducer and activator of transcription (JAK/STAT) pathway [153]. Suppresses inducible nitric oxide synthesis [154]
Rao et al. [141]	Detrimental	C57BL/6 mice	1.5% isoflurane with 25% oxygen balanced with nitrogen before ischemia	Induces HO-1 expression [155]. Preserves mitochondrial oxidative capacity [156]. Enhances the expression of guanosine triphosphate cyclohydrolase- (GTPCH-) 1 and eNOS [157]. Induces the generation of transforming growth factor-*β*1 (TGF-*β*1) [158]
Zhu et al. [142]	Detrimental	C57BL/6 mice	Rapamycin (1 mg/kg, i.p.) 1 hour prior to ischemia	Inhibits ERS [142]. Activates mTORC2/Akt pathway [138]. Recruits natural killer T (NKT) cells to IR region [159]. Activates JAK/STAT pathway, ERK, and eNOS [160]
Zhu et al. [138]	Detrimental	C57BL/6 mice	Rapamycin (1-5 mg/kg, i.p.) 1 hour prior to ischemia	Same as above

i.p.: intraperitoneal injection; i.v.: intravenous injection.

**Table 2:** The relationship between autophagy and liver IR injury.

Study	Effect of autophagy	Animal model Liver type	IR mode (ischemia/reperfusion time)	Autophagy change in IR	Interventions (effect on autophagy)	“Side effects” of intervention
Lee et al. [121]	Protective	BALB/c Lean	Warm 75% (45 min/2, 3, 6, 12, and 24 h)	Increase	Everolimus I (1 mg/kg each time, i.p.) 24 h before and immediately after reperfusion (+)	Reduces inflammation and apoptosis [121]. Reduces HO-1 expression and increases iNOS level [161].
Liu et al. [123]	Protective	SD Lean	Warm 75% (1 h/1 h, 6 h)	Increase	Baicalein (100 mg/kg, i.p.), 1 h prior to ischemia (+)	Activates HO-1 [123], PTEN/Akt/NO pathway [162]. Inhibits NF-*κ*B [163] and MAPK pathway [164].
Khader et al. [120]	Protective	C57BL/6 Lean	Warm 70% (1 h/12 h)	Increase	SRT1720 (20 mg/kg, i.v.) before reperfusion (+)	Stimulates the mitochondrial biogenesis. Reduces oxidative stress and inflammation [165].
Yang et al. [124]	Protective	C57BL/6 Lean/fatty	Warm 75% (1 h/0.5, 1.5, 3, 6, 12, and 24 h)	Increase	Tri-iodothyronine (0.002 mg, i.p.) precondition (+)	Reduces oxidative stress, apoptosis, and inflammation. Activates MEK/ERK/mTORC1 pathway [124].
Zhao et al. [166]	Protective	C57BL/6 Lean/fatty	Warm 75% (1 h/20 min)	Increase	Calpain inhibitor III (10 mg/kg, i.p.) 6 h prior to ischemia (+)	Inhibits the degradation of structural proteins. Suppresses apoptosis. Alters Ca^2+^ handling [167].
Li et al. [168]	Detrimental	C57BL/6 Lean	Warm 75% (1.5 h/2, 6, 12, and 24 h)	Increase	miR-17 agomir or antagomir (10 nM) 24 h prior to ischemia (+)	Inhibits PTEN [169], STAT3 [168], and death receptor 6 (DR6) [170].
Kang et al. [5]	Detrimental	C57BL/6 Lean	Warm 75% (1 h/1, 5, and 24 h)	Increase	Melatonin (10 mg/kg, i.p.) 15 min prior to ischemia and again immediately before reperfusion (-)	Inhibits oxidative stress. Improves the endothelial function. Restores mitochondrial function. Suppresses TLR and JNK pathways [94]. Activates RISK, SAFE, ERK1/2, PKB, PKC, JAK/STAT3, Sirt1/Sirt3, AMPK*α*, and Notch1/Mfn2 pathways [122].
Gotoh et al. [115]	Detrimental	Wistar Lean	Cold 100% (24 h/15 min)	Increase	Wortmannin (100 nM) or LY294002 (10 *μ*M) was added to the UW solution in the in situ perfusion and during storage, respectively (-)	Inhibits PI3K/Akt pathway [115].
Shen et al. [126]	Detrimental	BALB/c Lean	Warm 75% (45 min/4, 8, and 16 h)	Increase	Ethyl pyruvate (20 mg/kg, 40 mg/kg, and 80 mg/kg, i.v.) 1 h prior to ischemia (-)	Inhibits HMGB1/TLR/NF-*κ*B pathway [171]. Suppresses oxidative stress [172] and apoptosis [126].
Gupta et al. [125]	Detrimental	C57BL/6 Fatty	Warm 75% (20 min/24 h)	Increase	Ex4 (20 *μ*g/kg i.v.) 2 h prior to ischemia and immediately after surgery (-)	Activates Nrf2 [173], Akt/eNOS [174], and HMGB1 [175] pathways.
Yun et al. [176]	Protective	C57BL/6 Lean	Warm 75% (1 h/1, 4, and 24 h)	Decrease	Hemin (30 mg/kg) 16 h and 3 h prior to ischemia; carbon monoxide-releasing molecule-2 (20 mg/kg, i.p.) immediately before reperfusion (+)	Activates HO-1 [176], nuclear factor-erythroid 2-related factor 2 (Nrf2) [177]. Suppresses NF-*κ*B p65 nuclear translocation [177] and calpain-2 [176].
Kim et al. [178]	Protective	C57BL/6 Lean	Cold 100% (45 min/2 and 4 h)	Decrease	Carbamazepine (25 mg/kg, i.p.), overnight before IR (+)	Inhibits MPT Ca^2+^ overload. Suppresses calpain-2 [178].
Zaouali et al. [139]	Protective	Zucker Fatty	Cold 100% (24 h/2 h)	Decrease	Melatonin and trimetazidine were added to the UW solution during graft storage for 24h (+)	Same as above.
Minor et al. [179]	Protective	Wistar Fatty	Cold 100% (20 h/1.5 h and 2 h)	Decrease	Hypothermic reconditioning during the last 90 minutes of preservation (+)	Inhibits oxidative stress [179]. Enhances mitochondrial function [180].

i.p.: intraperitoneal injection; i.v.: intravenous injection.
